# Uptake of HPV vaccination among boys after the introduction of gender-neutral HPV vaccination in Germany before and during the COVID-19 pandemic

**DOI:** 10.1007/s15010-023-01978-0

**Published:** 2023-02-10

**Authors:** Cornelia Wähner, Johannes Hübner, Dörte Meisel, Jörg Schelling, Rebecca Zingel, Sarah Mihm, Regine Wölle, Miriam Reuschenbach

**Affiliations:** 1 Medical Affairs Department, MSD Sharp and Dohme GmbH, Munich, Germany; 2 Dr. Von Hauner Children’s Hospital of Ludwig Maximilian University, Munich, Germany; 3 Wettin Gynecological Practice, Wettin-Löbejün, Germany; 4 Medizinische Klinik IV, Hospital of Ludwig Maximilian University, Munich, Germany; 5 IQVIA Commercial GmbH and Co. OHG, Frankfurt, Germany; 6 Market Access Department, MSD Sharp and Dohme GmbH, Munich, Germany; 7 Global Medical and Scientific Affairs, MSD Sharp and Dohme GmbH, Levelingstrasse 4a, 81673 Munich, Germany

**Keywords:** Human papilloma virus, HPV vaccination, HPV immunization, Boys, Vaccination rate, Immunization rate, Germany

## Abstract

**Background:**

HPV vaccination has been recommended and reimbursed for girls in Germany since 2007. In June 2018 the German Standing Committee on Vaccination (STIKO) recommended the gender-neutral vaccination of adolescents aged 9 to 14 years with catch-up through age 17. Objectives of this study were to describe the uptake of vaccination in boys before and during the COVID-19 pandemic.

**Methods:**

The study used data from a proprietary electronic medical record database and a database with information on nationally dispensed vaccine doses. The monthly number of first doses of HPV vaccinations in boys and girls aged 9–17 years in the period from 01/2018 to 12/2021 was determined. In addition, for boys the cumulative vaccination rates were calculated for initiated and completed vaccination series.

**Results:**

Four months after the introduction of mandatory reimbursement for boys, the monthly numbers of first doses were comparable to that of girls. Compared to the same month in 2019, the number of first doses declined by up to 49% (girls) in 2020 and 71% (boys) in 2021. At the end of 2021, the vaccination rate for 15-year-old boys (2006 birth cohort) reached 44.4% for initiated and 26.4% for completed series.

**Conclusion:**

After an initial dynamic increase in HPV vaccinations in boys, the impact of COVID-19 was particularly strong in the second year of the pandemic. At the end of 2021 vaccination rates were still low. Efforts are needed to catch-up on adolescents that missed doses during the pandemic and to increase uptake.

## Introduction

Human papillomaviruses (HPV) are the most common sexually transmitted pathogens, with nearly 100% of all women and men contracting an HPV infection at least once in their life [1].

HPV infection can cause a number of diseases in women and men, including genital warts (condylomata acuminata) and various types of cancers [2]. HPV is the causative agent in almost all cervical cancers and causes approximately 85% of anal, 70% of vaginal, 40% of oropharyngeal and 30% of penile and vulvar cancers [3–5]. The most common disease caused by HPV in women in Germany is cervical cancer, accounting for around 4600 new cases per year [5]. According to estimates by the Center of Cancer Registry Data at the Robert Koch Institute (RKI), at least 1600 men also develop HPV-associated cancers every year. Most of these are oropharyngeal (approximately 750 cases), followed by anal (approximately 600 cases) and penile cancers (approximately 250 cases) [5]. A rise in cases of HPV-associated oropharyngeal cancers in particular has been observed in recent years [6].

HPV vaccination for girls – initially only for the 12–17-year-old age group with a 3-dose-schedule – has been recommended by the Standing Vaccination Committee (STIKO) since 2007. Since 2014 the recommendation has included standard vaccination for girls aged 9–14 (2 doses in months 0 and 6–12) and catch-up vaccination for girls aged 15–17 (3 doses in months 0, 2, 6). Both vaccination schedules should be completed within a year. [5]. From 2011 to 2019 the rate for a full vaccination series in 15-year-old girls rose from 27.2% to 47,2% [7]. With not even 50% fully vaccinated 15-years old girls the HPV vaccination rate in Germany is at low level. Possible reasons are not studied but are likely multi-factorial including low awareness and acceptance of HPV as a routine vaccine, no organized easy access in this age group and safety concerns. In this age range HPV is the only standard vaccination in Germany except of booster vaccinations for tetanus, diphtheria, polio, pertussis for which no vaccination rates are published. Therefore, a direct comparison of HPV vaccination rates with those of other adolescent vaccinations is not possible. Overall, however, it can be noted that children in Germany are often vaccinated too late and too little, although not to the extent of HPV. For example, in 2019, only 75,6% of children received a complete vaccination scheme of measles vaccination (2 doses at the age of 11 and 15 months) and 90.2% of children were vaccinated with the triple vaccination against diphtheria, tetanus and pertussis at the age of 15 months. As a result, national and international vaccination goals are not reached [7].

In June 2018 the STIKO extended the HPV vaccination recommendation to boys in the same age groups [5]. This decision was based chiefly on the following considerations [5]:

1. The high burden of disease of HPV-associated cancers in men
2. The proven efficacy and safety of HPV vaccination in men
3. The estimated positive epidemiological impact on HPV-associated cancer case numbers in Germany, especially given the low vaccination rate for girls and the resulting still moderate level of herd immunity
4. Gender equality

Based on the decision of the Federal Joint Committee (G-BA) of September 20, 2018, HPV vaccination has been reimbursed as a compulsory benefit of all the statutory health insurance schemes for both sexes aged 9–17 since January 2019 [8].

Generally only physicians are permitted to vaccinate in Germany and there is no general invitation or reminder system. Exceptions only exist for COVID-19 vaccinations [9] as well as influenza vaccinations within some pilot projects where pharmacists are allowed to vaccinate. Vaccinations, including HPV are provided in physician offices, only small school-based vaccination pilot projects were done for HPV [10]. For HPV vaccination specifically this means that pediatricians, GPs and gynecologists are the main vaccinators because they see patients of the target age group in their offices. The physician or the parent needs to actively raise the need for HPV vaccination once the child becomes eligible making the system extremely opportunistic. According to STIKO all available licensed products can be used. In 2006 the quadrivalent vaccine (immunization against HPV6, 11, 16 and 18) was first licensed and available in Germany, followed by the bivalent (immunization against HPV16 and 18) in 2007 and the nonavalent vaccine (immunization against HPV6, 11, 16, 18, 31, 33, 45, 52 and 58) in 2016. During the course of the study, the bivalent and the nonavalent vaccines were available in Germany.

To our knowledge, this is the first study to examine the uptake of HPV vaccination among boys based on the monthly number of vaccinations in Germany, including the dynamic during the COVID-19 pandemic. The primary aim of the study was to describe the nationwide number of administered monthly first doses of HPV vaccinations in boys aged 9–17 for 01/ 2018 to 12/2021 to observe the dynamic of the vaccination uptake and compare those data to those of girls. The monthly breakdown of administered doses is an outcome that is not reported from other data sources but is a unique tool to assess short-term uptake dynamics, particularly during COVID-19. The evaluation was stratified according to the old (former West Germany) and new (former East Germany, German Democratic Republic) federal states of Germany, relevant medical specialist groups and co-administration of other vaccines with the HPV vaccine. To also consider the size of the eligible population and to make results comparable to other national and international reports, our secondary aim was the calculation of the cumulative vaccination rates for boys as a measure of population-wide coverage by male birth cohort.

## Methods

### Study design and data source

The study is a retrospective cohort study based on two proprietary databases: IQVIA™ Vaccine Analyzer (hereafter called Vaccine Analyzer) and IQVIA™ PharmaScope Vaccine DocSplit (hereafter called PharmaScope). The Vaccine Analyzer database has been previously shown to be representative and suitable for pharmacoepidemiologic studies by Ohl et al. [11]. In this previous publication, the database Vaccine Analyzer has been described in detail: It contains anonymous information about diagnoses, prescriptions including administered vaccinations and demographic characteristics of patients of office-based specialists and general practitioners in Germany. Back data is available until 2014. Demographics such as age and gender distributions as well as vaccination rates for children, adolescents and adults have been computed and compared with official national data for Germany. In general, the study showed a good consistency between the Vaccine Analyzer and the official data for Germany by age and gender, especially for vaccinations in children [11].

The Vaccine Analyzer identifies HPV vaccinations administered in a nationally representative panel of 460 office-based physicians from 353 practices in the specialist groups of general practitioners and internists (hereafter referred to as “general practitioners”), pediatricians and gynecologists. Information is collected on the patient’s gender, year of birth, date of vaccination, vaccine product administered, co-administration of other vaccines, physician’s specialty and region of the practice (East, including Berlin and West, excluding Berlin). Additionally, an anonymized patient ID is generated for each patient. For the analysis of co-administrations, only those vaccines (tetanus, diphtheria, polio, pertussis) were selected for which the National Advisory Committee “STIKO” recommends boosters for the respective age group which are also those vaccines for which data on concurrent administration exist according to the Summary of Product Characteristics (SmPCs) of the European Medicines Agency (EMA).

The nation-wide numbers were then extrapolated utilizing the second database (PharmaScope) together with the distribution per month observed in the Vaccine Analyzer for stratifications by gender, age group, and other the variables. The PharmaScope database provides information on the number of nationally prescribed doses within the statutory and private health insurance sector in Germany without information on gender, age or other variables: For the doses reimbursed through statutory health insurances (relevant for approximately 85% of the German population) data from all relevant German pharmacy data processing centers are captured (so called “Sprechstundenbedarf”). For doses reimbursed by private health insurances (relevant for the remaining approximately 15% of the German population) individual prescription data from a panel of approximately 4500 pharmacies (~ 23% of all pharmacies in Germany) are used and extrapolated to the total number of pharmacies in Germany, taking into account certain characteristics. Pharmacies are stratified into 77 clusters, partially without geographical adjacency. Those 77 clusters are defined by sociodemographic homogeneity, purchasing power, population structure and insurance status and other factors and separated by, e.g., metropolitan from provincial areas in Germany. Additionally, 3 pharmacy turnover-size classes are defined (S, M, L): S constitutes 30% of the total national sales potential, M the middle next 30%, L the top last 40%.

The terms “patient”, “physician”, “medical practice”, and “pharmacy” used in connection with the data refer to anonymous, not personal, information pursuant to relevant data-protection regulations.

### Study population

In accordance with the vaccination age recommended by STIKO, the analysis included 9–14 and 15–17-year-old male and female adolescents born between 2000 and 2012 who received at least one HPV vaccine dose during the study period (January 2018 to December 2021). Since the database does not contain information on the exact date of birth, the vaccinees’ age was calculated as the year of the respective dose (first or last dose for completed series) minus the year of birth.

Bivalent and 9-valent vaccines (manufactured by GSK and MSD, respectively) were available in Germany during this time.

A dose was classified as first dose if no other dose was recorded in the database within 18 months before the vaccination date. According to the vaccine product labels and STIKO, the vaccination series should be completed within 12 or 13 months (depending on 2-or 3-doses scheme), so the 18-month window provided an additional buffer period. Vaccination series completion was defined as having 2 (for 9–14-year-olds) or 3 (for 15–17-year-olds) doses observed within the study period. It is possible for someone to have received a dose in a practice outside of the utilized panel, which would not be captured in the Vaccine Analyzer database, resulting in some misclassification. Additionally, because of the missing exact age some misclassification may occur for 15-year-old individuals. The birth cohort turning 15 in a given year was counted to the 9–14-year-old age group and thus 2 doses were considered as series completion, although they may have actually been already 15 when they received their first dose and consequently required 3 doses for completion.

### Data analysis

For the primary study objective, the nation-wide number of administered first HPV vaccination doses was calculated for each month between January 2018 and December 2021 using monthly numbers of doses from the Vaccine Analyzer database and applying those to the nation-wide number of dispensed vaccine doses in the PharmaScope database. Thereby, nation-wide estimates on administered doses stratified by gender, age (9–14 years, 15–17 years), specialist group (general practitioner, pediatrician, gynecologist), and region (West excluding Berlin vs. East including Berlin) were generated. In addition, the frequency of co-administration with tetanus, diphtheria, pertussis and polio vaccinations was analyzed. This study focused on descriptive statistical analyses of the available data.

For the secondary objective, the cumulative vaccination rates in boys, were estimated per birth cohort for the observation periods 2018, 2018–2019, 2018–2020 and 2018–2021. For those vaccination rates, the number of patients with a documented full series of vaccinations in the Vaccine Analyzer was extrapolated to national level with the aid of PharmaScope. The vaccination rates were calculated as the ratio of extrapolated vaccinated patients and the German population number based on the German Federal Statistical Office. Because of the time lag in the publication of the annual population figures by the Federal Statistics Office, the population estimates for 2018 were the denominator for all observation periods.

While monthly numbers of vaccines administered to girls were included in the analysis, the cumulative vaccination rates for girls were not calculated in the present analysis because, unlike boys, girls had already been eligible for vaccinations for several years before the start of the study, and the size of girls already vaccinated before the study period and the size of still unvaccinated girls was not known in the study population.

### Ethics

This study was conducted according to Guidelines for Good Pharmacoepidemiology Practices. The utilized databases contain only anonymized data and address all data protection regulations in Germany. Therefore, no ethics review was needed by an independent ethics committee or institutional review board.

## Results

### Monthly number of first doses of HPV vaccination in girls and boys in Germany after the recommendation and cost reimbursement for boys

**Fig. 1:**
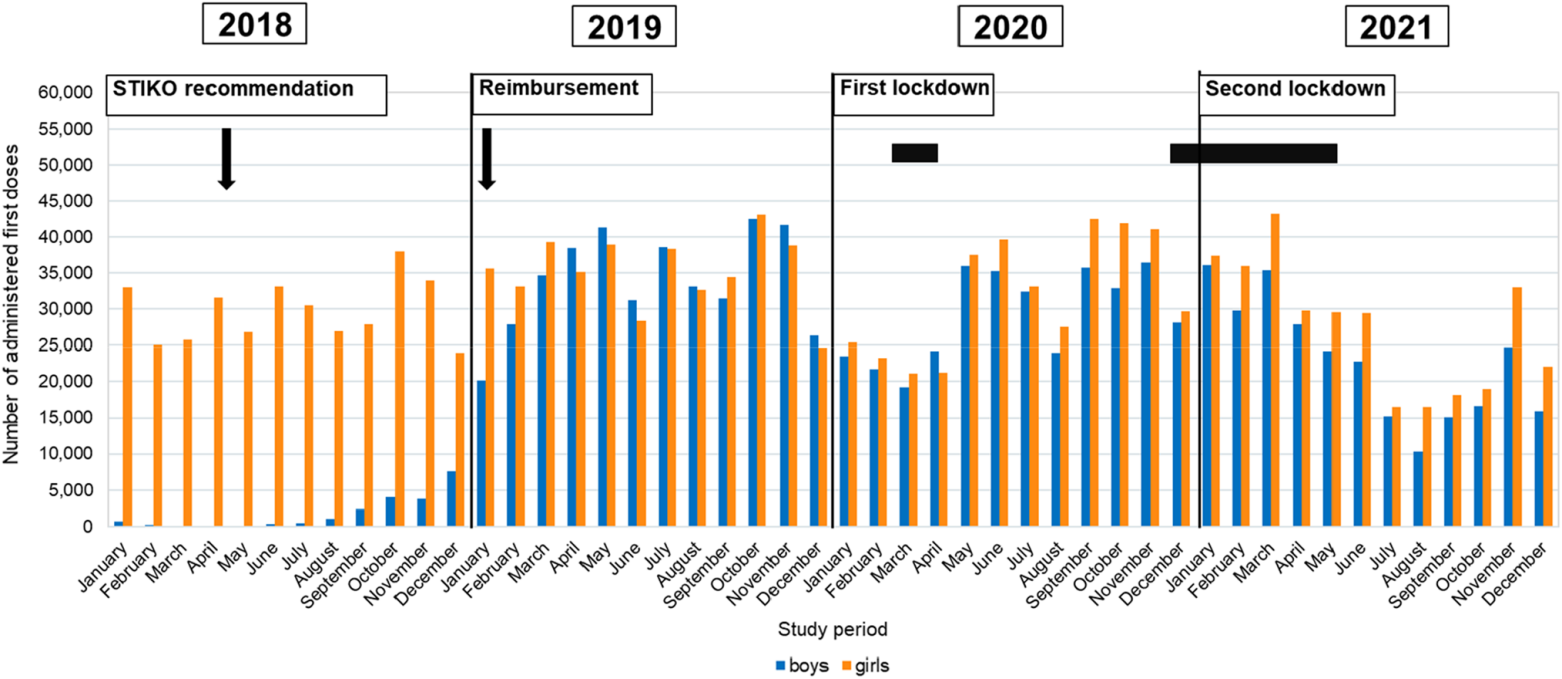
Number of monthly first doses of HPV vaccination in boys and girls aged 9–14 years in the 2018–2021 study period

**Fig. 2:**
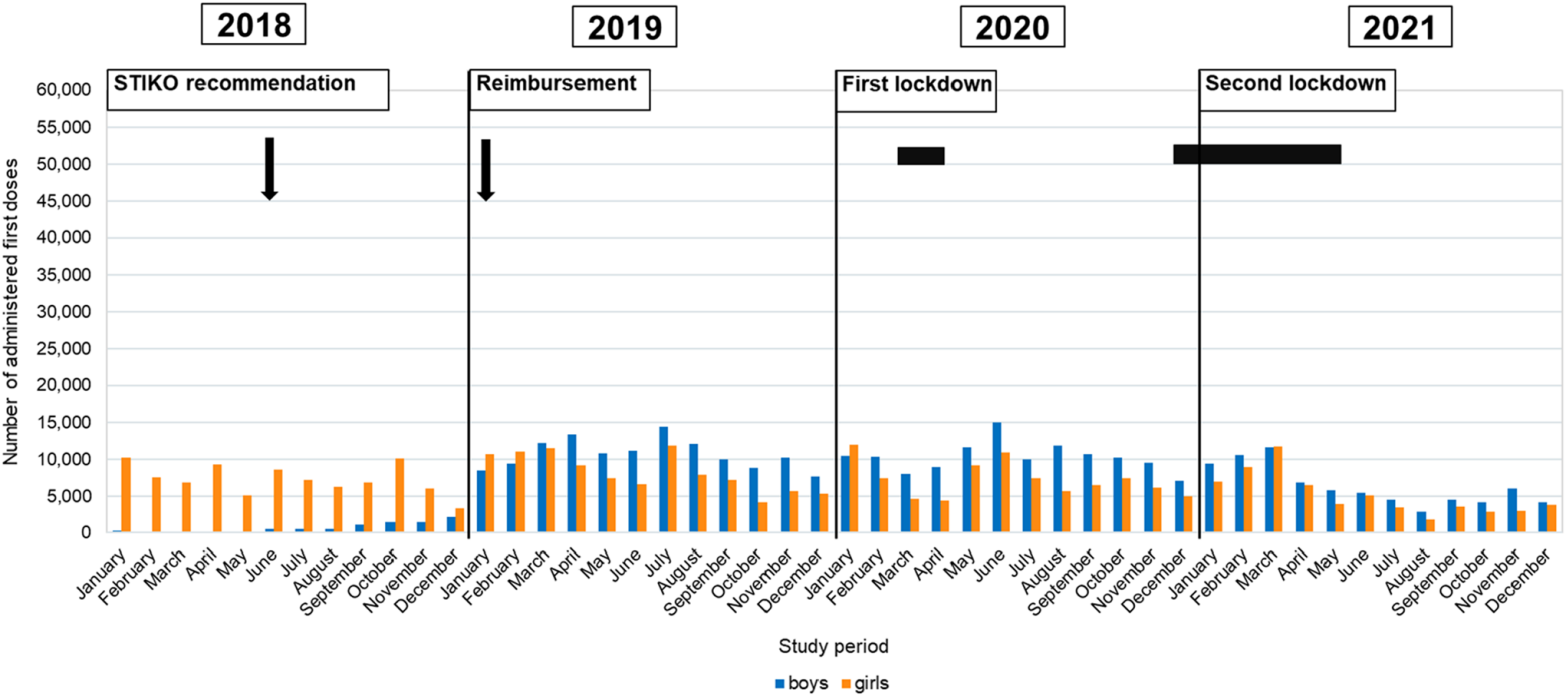
Number of monthly first doses of HPV vaccination in boys and girls aged 15–17 years in the 2018–2021 study period

In each month between January and May 2018, between 49 and 681 boys in the 9–14 age group and between 0 and 268 boys in the 15–17 age group across Germany received a first dose of HPV vaccination. The monthly number of first doses of HPV vaccination in girls between January and May 2018 was 25,028–33,009 (9–14 years) and 5123–10,257 (15–17 years). After the STIKO recommendation for boys was issued (June 2018), the number of monthly HPV vaccinations in boys increased from 366 to 7560 in the 9–14 age group and from 466 to 2110 in the 15–17 age group between June and December 2018 (Figs. 1, 2).

In the first month of reimbursement for HPV vaccinations for boys (January 2019), the number of first doses of vaccination in the 9–14 age group increased by a factor of 2.7 compared to the previous month (Fig. 1). In the age group of 15–17-year-old boys, primary vaccinations quadrupled compared to the previous month (Fig. 2). Four months (9–14 years) and three months (15–17 years) after the introduction of mandatory reimbursement, the number of monthly first doses of vaccination in boys reached the same level as that for girls. In the following months up to December 2019, a comparable number of boys and girls in the 9–14 age group received a first vaccination (Fig. 1), whereas in the 15–17 age group, the number of first doses of HPV vaccination among boys exceeded that of girls as of March 2019 (Fig. 2).

**Fig. 3:**
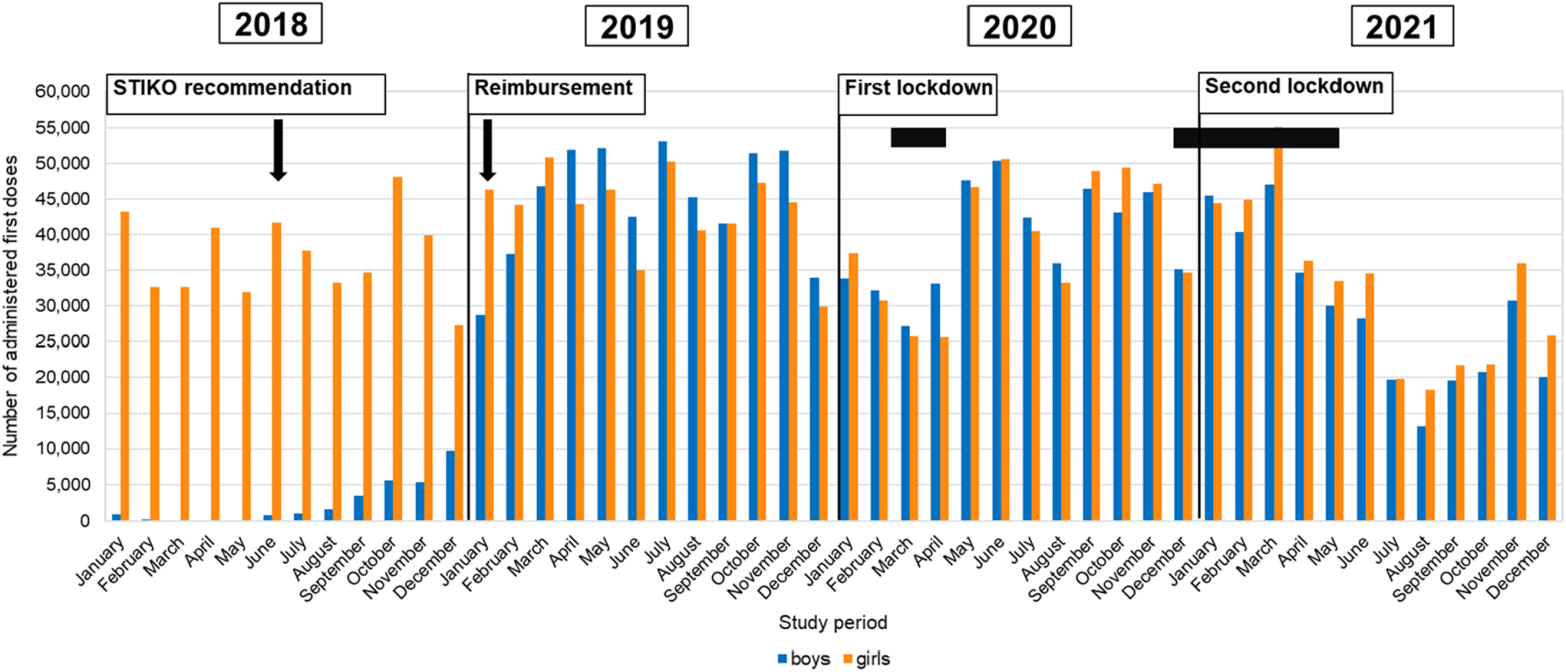
Number of monthly first doses of HPV vaccination in boys and girls aged 9–17 years in the 2018–2021 study period

Figure 3 shows the monthly number of first doses of vaccination for the entire cohort of 9–17-year-old girls and boys for the entire study period 01/2018–12/2021.

### Monthly number of first doses of HPV vaccination during the COVID-19 pandemic

With the onset of the COVID-19 pandemic and the first lockdown, early 2020 saw an initial decline in first doses of HPV vaccination among boys and girls, particularly in the 9–14 age group. Numbers began to rise again from May 2020, leveling off at between 24,000 and 43,000 (depending on gender) over the entire year up to March 2021 (Fig. 1).

**Fig. 4:**
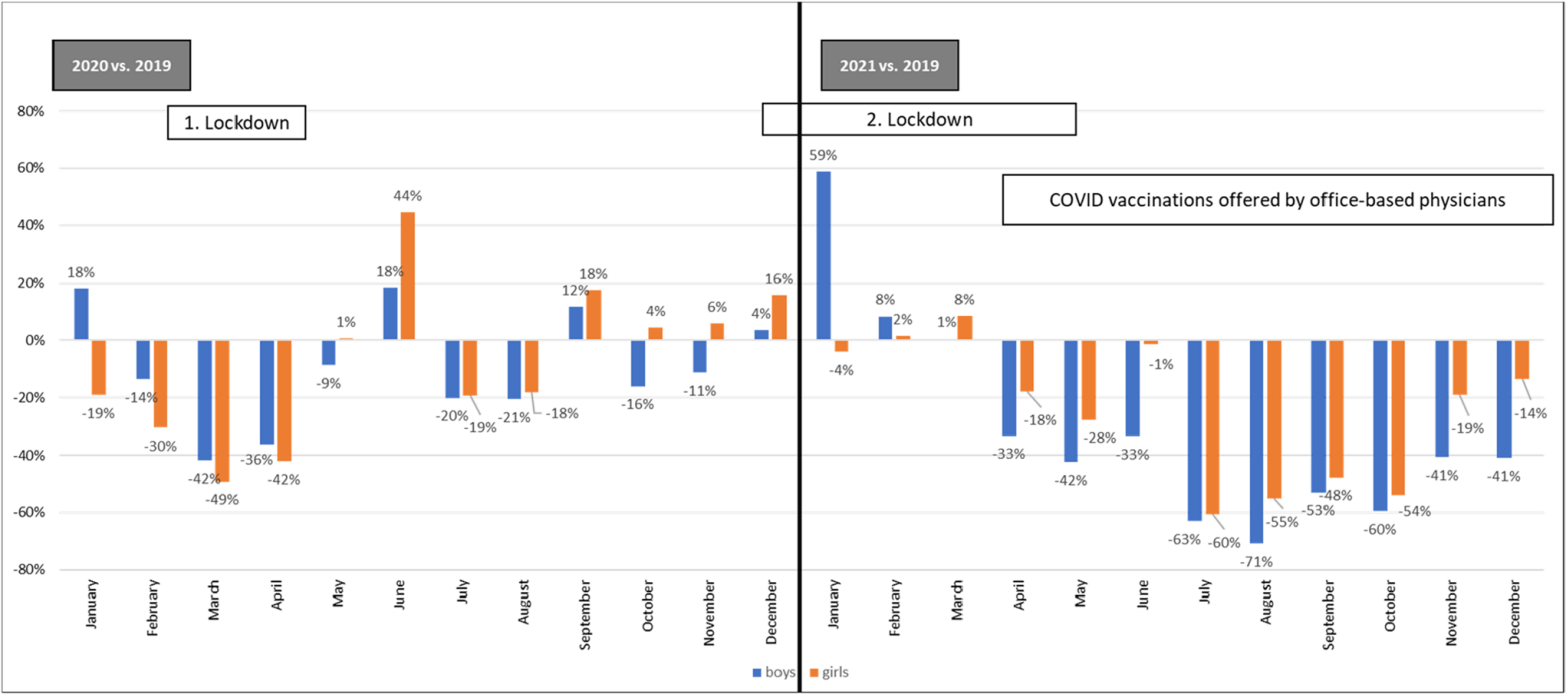
Rise/decline of first doses of HPV vaccination in boys and girls (9–17 years) in 2020/2021 compared to 2019

Looking at the entire age cohort of 9–17-year-olds, a continuous steep decline in monthly administered doses occurred from April 2021, reaching a minimum of 13,178 first doses for boys and 18,261 for girls in August 2021 (Fig. 3). This decrease resulted in a maximum decline of − 71% for boys (9–17 years) in August 2021 and − 60% for girls (9–17 years) in July 2021 compared to the same months of 2019 (Fig. 4). In the months that followed and up to the end of the study period in December 2021, the number of first doses of vaccination in boys and girls gradually rose again (Fig. 3).

### Specialist group distribution for first doses of HPV vaccination and co-administration with other vaccines

**Fig. 5:**
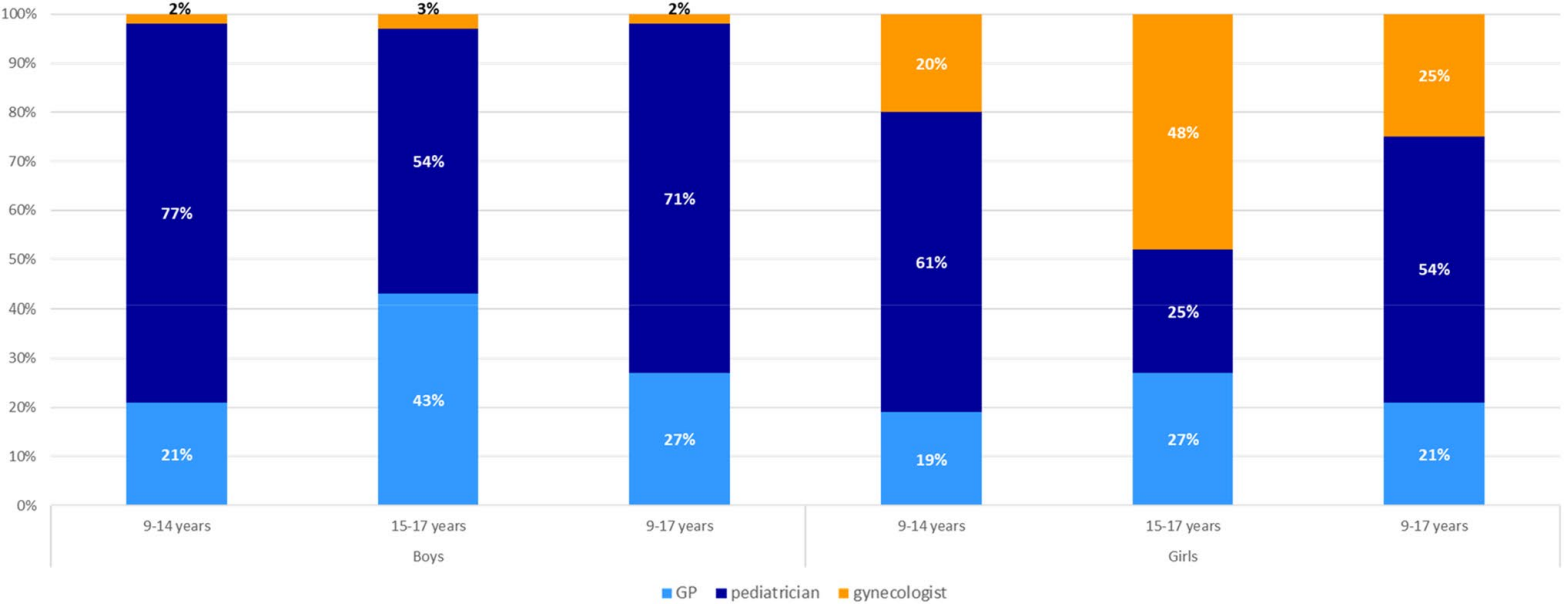
Distribution among specialist physician groups for first doses of HPV vaccination by gender and patient age group in the 2018–2021 study period

The majority of the vaccinated boys and girls in the 9–17 age group received their first HPV vaccination from a pediatrician (71 and 54%, respectively). Looking at the younger and older age groups, 21% of the girls and 19% of the boys in the 9–14 age group and 43% of the boys and 27% of the girls in the 15–17-year age group received their first vaccination from a general practitioner. 25% of the 9–17-year-old girls were vaccinated by a gynecologist (Fig. 5).

Among 9–17-year-old boys who were vaccinated against HPV, 27.1% received an additional dose of another vaccine on the same day (9–14 age group: 32.4%; 15–17 age group: 14.9%). The comparative figures for girls aged 9–17 was 23.7% (9–14 age group: 26.3%; 15–17 age group: 14.9%) (Table 1).

**Table 1:** Co-administrations on the same day as the HPV vaccination

Co-administrations 2018–2021						
	Boys			Girls		
	9–14 years (%)	15–17 years (%)	9–17 years (%)	9–14 years (%)	15–17 years (%)	9–17 years (%)
Tdap	5.0	4.9	5.0	4.4	3.9	4.3
Td-IPV	0.1	0.3	0.2	0.2	0.6	0.2
Tdap-IPV	27.3	9.7	21.9	21.7	10.4	19.2
Total (with co-administration)	32.4	14.9	27.1	26.3	14.9	23.7

### HPV vaccination rates for boys in Germany

Cumulative vaccination rates for first doses of vaccination and completed vaccination series were calculated from the monthly administered vaccination doses for the individual birth cohorts of 9–17-year-old boys for the observation periods 2018, 2018–2019, 2018–2020 and 2018–2021.

**Fig. 6:**
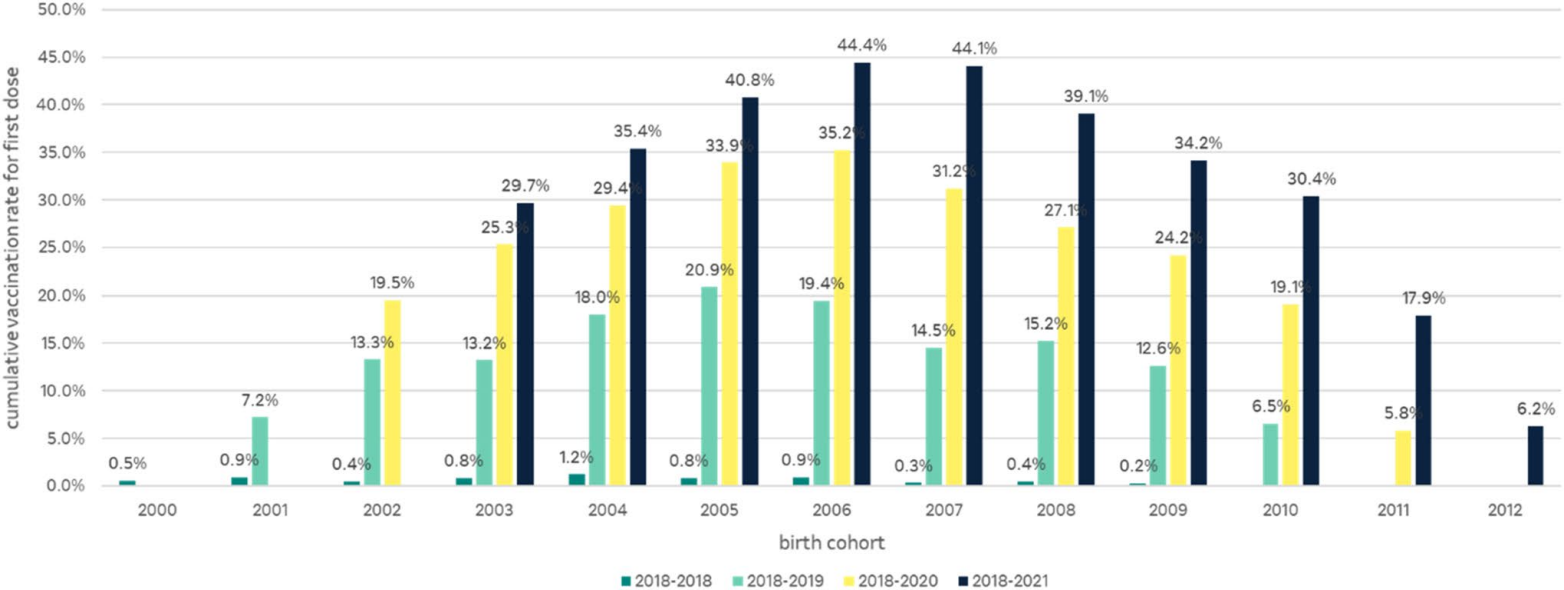
Cumulative HPV vaccination rates for first doses in boys by cohort for the 2018, 2018–2019, 2018–2020 and 2018–2021study periods

As a proportion of the overall male population of corresponding age, the vaccination rates for the first doses of HPV vaccination in 2018, the year the recommendation was issued, was between 0.2% (2009 cohort, 9 years old at the end of 2018) and 1.2% (2004 cohort, 14 years old at the end of 2018). By the end of 2019 the vaccination rates (2018–2019) for first doses increased to values between 6.5% (2010 cohort, 9 years old at the end of 2019) and 20.9% (2005 cohort, 14 years old at the end of 2019). End of 2020, the first year of the COVID-19 pandemic, the vaccination rates (2018–2020) ranged between 5.8% (2011 cohort, 9 years old) and 35.2% (2006 cohort, 14 years old). At the end of the study period in December 2021, the cumulative vaccination rates for first doses for boys was between 6.2% (2012 cohort, 9 years old) and 44.4% (2006 cohort, 15 years old) (Fig. 6).

**Fig. 7:**
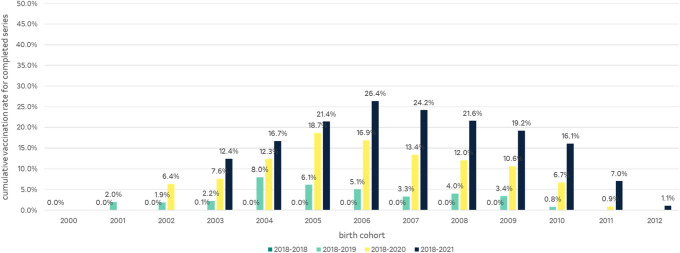
Cumulative HPV vaccination rates for a full vaccination series in boys per cohort for the study periods 2018, 2018–2019, 2018–2020 and 2018–2021

In 2018, the vaccination rate for completed series across all age groups of boys was close to 0.0%. At the end of 2019 (2018–2019) between 0.8% (2010 cohort, 9 years old) and 8.0% (2004 cohort, 15 years old) and at the of 2020 (2018–2020) between 0.9% (2011 cohort, 9 years old) and 18.7% (2005 cohort, 15 years old) of boys completed a full series of HPV vaccination. Between 1.1% (2012 cohort, 9 years old) and 26.4% (2006 cohort, 15 years old) of boys received a full HPV vaccination series by the end of the study period (December 2021) (Fig. 7).

**Fig. 8:**
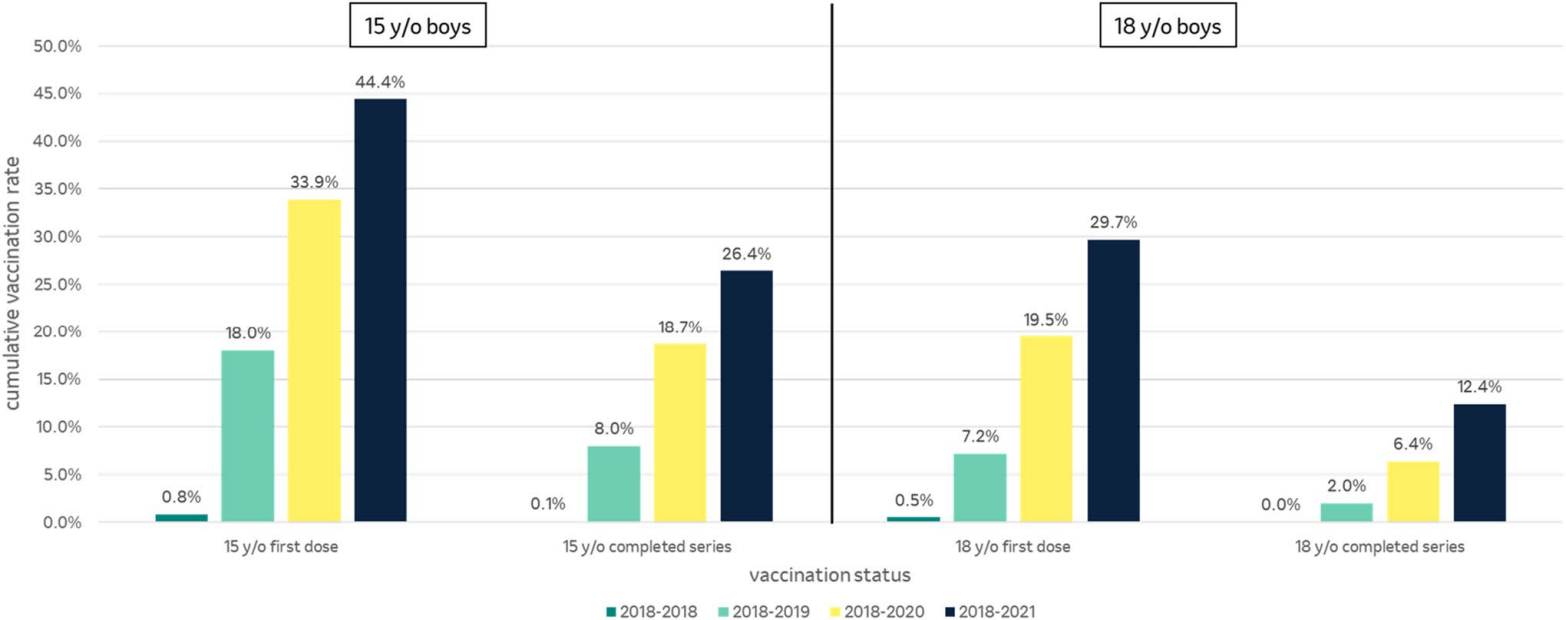
Cumulative HPV vaccination rates for first doses and full vaccination series for 15- and 18-year-old boys for the study periods 2018, 2018–2019, 2018–2020 and 2018–2021. The exact age of the vaccinees is not known. For 15-year-olds/18-year-olds, the 2003/2000 cohorts are shown for 2018, the 2004/2001 cohorts for 2018–2019, the 2005/2002 cohorts for 2018–2020 and the 2006/2003 cohorts for 2018–2021

Because the STIKO recommendation discriminates between a standard vaccination with a 2-dose scheme through age 14 and a catch-up vaccination with a 3-dose scheme through age 17, the vaccination rates for 15-year-olds (2003–2006 cohort) and 18-year-olds (2000–2003 cohort) were especially looked at. In 2018, the year of the recommendation, few first doses of HPV vaccination were given in both age groups: 0.8% in 15-year-olds and 0.5% in 18-year-olds. At the end of 2019, 18.0% of 15-year-old boys and 7.2% of 18-year-old boys received their first HPV vaccination. By the end of 2020, 33.9% of 15-year-old and 19.5% of 18-year-old boys had started an HPV vaccination series, and by the end of 2021 those figures had risen to 44.4 and 29.7%, respectively (Fig. 8). A full vaccination series was completed by 8.0% of 15-year-old and by 2.0% of 18-year-old boys by the end of 2019, and by 18.7% of 15-year-old and 6.4% of 18-year-old boys by the end of 2020, rising to 26.4 and 12.4%, respectively, by the end of the study period in 2021 (Fig. 8).

It is to be noted, that the rates for both first doses and full vaccination series were generally higher in the new states than in the old states of Germany (Tables 2 and 3).

**Table 2:** Cumulative vaccination rates by year, region and birth cohort for the first dose of vaccination

First vaccination, cumulative	2018–2018			2018–2019			2018–2020			2018–2021		
	Former FRG (%)	Former GDR (%)	National (%)	Former FRG (%)	Former GDR (%)	National (%)	Former FRG (%)	Former GDR (%)	National (%)	Former FRG (%)	Former GDR (%)	National (%)
Birth cohort 2000	0.5	0.7	0.5									
Birth cohort 2001	0.8	1.1	0.9	7.0	8.4	7.2						
Birth cohort 2002	0.3	0.8	0.4	12.9	15.4	13.3	18.9	22.6	19.5			
Birth cohort 2003	0.7	1.1	0.8	12.6	16.3	13.2	24.7	28.2	25.3	29.1	32.5	29.7
Birth cohort 2004	1.2	1.0	1.2	17.5	20.2	18.0	28.4	34.1	29.4	34.3	40.2	35.4
Birth cohort 2005	0.7	1.2	0.8	20.3	23.7	20.9	33.3	36.5	33.9	40.7	41.0	40.8
Birth cohort 2006	0.9	0.9	0.9	19.2	20.4	19.4	34.9	36.9	35.2	44.7	43.5	44.4
Birth cohort 2007	0.3	0.3	0.3	14.1	16.0	14.5	30.8	32.7	31.2	43.9	44.9	44.1
Birth cohort 2008	0.4	0.4	0.4	14.7	17.5	15.2	26.0	32.0	27.1	38.1	43.0	39.1
Birth cohort 2009	0.2	0.3	0.2	12.2	14.6	12.6	23.4	27.4	24.2	33.6	36.4	34.2
Birth cohort 2010				6.1	8.0	6.5	18.9	19.7	19.1	30.3	30.7	30.4
Birth cohort 2011							5.4	7.4	5.8	17.6	19.1	17.9
Birth cohort 2012										6.3	5.9	6.2

*FRG* Federal Republic of Germany = old states of Germany, *GDR* German Democratic Republic = new states of Germany

**Table 3:** Cumulative vaccination rates by year, region and birth cohort for the full vaccination series

Fully vaccinated, cumulative	2018–2018			2018–2019			2018–2020			2018–2021		
	Former FRG (%)	Former GDR (%)	National (%)	Former FRG (%)	Former GDR (%)	National (%)	Former FRG (%)	Former GDR (%)	National (%)	Former FRG (%)	Former GDR (%)	National (%)
Birth cohort 2000	0.0	0.0	0.0									
Birth cohort 2001	0.0	0.0	0.0	1.9	2.3	2.0						
Birth cohort 2002	0.0	0.0	0.0	1.8	2.6	1.9	6.3	6.9	6.4			
Birth cohort 2003	0.1	0.1	0.1	1.9	3.8	2.2	7.2	9.6	7.6	12.0	14.1	12.4
Birth cohort 2004	0.0	0.0	0.0	7.6	9.6	8.0	11.6	15.8	12.3	15.6	21.7	16.7
Birth cohort 2005	0.0	0.0	0.0	6.0	7.0	6.1	18.3	20.5	18.7	20.9	23.6	21.4
Birth cohort 2006	0.0	0.0	0.0	5.0	5.3	5.1	16.7	17.5	16.9	26.5	26.0	26.4
Birth cohort 2007	0.0	0.0	0.0	3.2	4.2	3.3	13.0	14.9	13.4	24.1	24.5	24.2
Birth cohort 2008	0.0	0.0	0.0	3.8	4.8	4.0	11.4	14.4	12.0	21.1	23.6	21.6
Birth cohort 2009	0.0	0.0	0.0	3.1	4.6	3.4	10.0	13.0	10.6	18.7	21.0	19.2
Birth cohort 2010				0.7	1.2	0.8	6.4	8.3	6.7	15.8	17.0	16.1
Birth cohort 2011							0.7	1.6	0.9	6.6	9.0	7.0
Birth cohort 2012										1.1	1.0	1.1

*FRG*  Federal Republic of Germany = old states of Germany, *GDR*  German Democratic Republic = new states of Germany

## Discussion

This is the first study to describe the monthly uptake of first doses of HPV vaccination after the introduction of the STIKO recommendation and cost reimbursement for boys as well as the dynamics during the COVID-19 pandemic. In addition, the HPV vaccination rate among boys in Germany is examined from the time of the recommendation in 2018 until the end of 2021.

The results of the study show that just four months after the introduction of reimbursement for HPV vaccination in boys, the monthly vaccination level for first doses of vaccination was comparable to that of girls and exceeded it in some of the subsequent months. It should be noted that the total number of unvaccinated boys was greater than that of girls, for whom reimbursement has been in place for several years, and the demand for boys was therefore higher especially shortly after the recommendation and implemented reimbursement. At the end of 2021, the vaccination rate for 15-year-old boys (2006 cohort) reached 44.4% for first doses of vaccination and 26.4% for a full vaccination series. The number of HPV vaccine doses administered per month fell substantially among German adolescents during the COVID-19 pandemic (2020/2021 compared to the same months in 2019). In 2020, the biggest declines were seen during the first lockdown. The dip in first doses of HPV vaccinations was even greater in 2021, with the monthly uptake falling short of the 2019 figures by up to 71% for boys and 60% for girls. While initially COVID-19 vaccinations for adults were provided largely in public COVID-19 immunization centers, since April 2021 COVID-19 vaccinations were also offered by most office-based physicians. In June 2021 STIKO published a COVID-19 vaccination recommendation also for children and as for adults, vaccinations were provided by office-based physicians in addition to immunization centers. Initially the recommendation was only for children of high risk 12–17 years old, in August for all children 12–17 years old and since December 2021 also for children 5–11 years old [12]. It is assumed that the provision of COVID-19 vaccinations in doctor’s practices in 2021 contributed to the observed decline in HPV vaccinations in that it resulted in routine vaccinations being postponed either by the physicians or the parents. Data from a relevant health insurance company (Deutsche Angestellten Krankenkasse, DAK) showed a decline in physician visits during the pandemic. Among children and adolescents aged 0–17 insured by DAK, there were 4% fewer outpatient physician visits in 2021 compared to 2019. Extrapolated to the national level, COVID-19 led to 1.3 million fewer physician visits in the age cohort of 0–17-year-olds [13].

Vaccination rates for both, first doses and full vaccination series were generally higher in the new federal states (former GDR) than in the old federal states of Germany (former West Germany). This is in line with HPV vaccination reports from the Robert-Koch-Institute [7]. It points towards a higher acceptance or better organization of HPV vaccination in the new states but needs more research to understand concrete drivers. Interestingly, not all vaccines seem to have better coverage in the new states and for example COVID-19 vaccination coverage is even lower in the new states than in the old states [7].

Initial data from the vaccination surveillance by the RKI have so far only been published for 2018 and 2019 and show HPV vaccination rates of 5.1 and 2.5% for complete vaccination series for 15- and 18-year-old boys, respectively, at the end of 2019 [14] and hence are comparable to our results for the same years.

For girls, the costs of the HPV vaccination were covered for the first time in 2007 for 12–17-year-olds, and in a survey later that year 23.8% of the girls in this age group reported having received at least one HPV vaccination [15]. At first glance, these historical figures suggest that the initial uptake of vaccinations may have been slightly better among girls than is currently the case among boys. However, a direct comparison of the implementation dynamics between boys and girls is not possible, as major differences exist in terms of the time elapsed between the STIKO recommendation and mandatory reimbursement, the vaccination age and the data collection method. In the second year of cost reimbursement (2008), 32.2% of 12-to-17-year-old girls had received at least one HPV vaccine dose [16]. Looking at the rate for complete vaccination series in boys at the end of the study period, it is noteworthy that 40% of the 15-year-olds and 58% of the 18-year-olds had not completed a full series of vaccinations. Comparable surveillance data on completion from the RKI are not yet available for boys. However, looking at the completion rates for 15- and 18-year-old girls, it is observed that 24.8 and 20.7%, respectively, did not complete an HPV vaccination series that they had started in 2019 [14].

It is striking that the percentage of boys who did not complete an initiated vaccination series is almost twice that of girls if comparing our data with those official figures from girls. This might be due to the COVID-19 pandemic. Another reason for this gap in series completion might be the limitation of the data source: the results can only provide a snapshot with regard to completion, since the study period did not always allow a full vaccination series to be given, e.g., people administered dose 1 in 2nd half of 2021 may not be eligible for dose 2 in the same year. Moreover, there is the methodological limitation that the Vaccine Analyzer does not allow patients to be tracked across different medical practices, so that the data do not reflect the completion of a vaccination series in cases where patients changed practices. It is reasonable to surmise that this affects mainly the older age group in which patients transitioned from a pediatrician to general practitioner. Nevertheless, those potential methodological limitations inherent to the Vaccine Analyzer can likely be considered as minor because the methodology has been validated against the official surveillance data from the RKI for other vaccines and calendar periods and good agreement has been found [11].

The results of the study also provide insights into the implementation of HPV vaccination in terms of the vaccinating physician’s specialty and co-administered other vaccines on the day of HPV vaccination. For both sexes, we found that pediatricians cover the majority of HPV vaccinations, mainly in the younger cohort of 9–14-year-old children with 61% of first doses in girls and 77% of first doses in boys. But particularly in the older age group of 15–17-year-old adolescents we show that general practitioners and in girls also gynecologists cover a significant amount of HPV vaccinations. To not miss older children before they drop out of reimbursement, it is important that general practitioners and gynecologists are aware of their key role and implement HPV vaccinations in their practice accordingly. This may be particularly important now to catch-up on those that missed doses during the COVID-19 pandemic. Another important finding is that approximately only a quarter of HPV vaccinations in the study population were given concomitantly with Tdap-IPV vaccines. It may be worth exploring whether appointments for other vaccinations may be more often used also for HPV vaccinations, as long as in line, with the product’s label.

From an international point of view, the published German HPV vaccination rates for girls are generally at a low level. Germany only ranks 17^th^ among 25 European countries and is nowhere near to reaching the WHO’s 90% target for girls up to the age of 15 until the year 2030 [10, 17, 18]. Higher vaccination rates have also been published for boys in other countries than observed in our study. The reasons for the poor uptake in Germany are not well studied, but it is likely that there are several factors that contribute: (1) Awareness about HPV and HPV vaccination. In a population-based survey 64% of adults in Germany had heard about HPV, but only 45% of those knew that there are vaccines available to prevent HPV [19]. Hence, increasing awareness among the public seems to be one opportunity to improve coverage. However, it should be noted that health care providers have a key role in making their clients aware about HPV vaccination. Research in the US has shown that a strong and presumptive provider recommendation increases HPV vaccine acceptance and uptake significantly [20] and more and better communication training for health care personnel in Germany could be proposed at least to a similar extent as public awareness campaigns. (2) Vaccine confidence, hesitancy, and safety concerns. There are no HPV-specific data for Germany available, but according to a vaccine hesitancy survey in 17 European countries, Germany ranked in the positive upper quarter on place 13 with more than 60% of parents classified as not at all vaccine hesitant (this percentage ranged in the 17 countries from 23 and 85%) [21]. According to another study in Western Europe that looked at vaccine hesitancy and populist politics less than 10% of the German population would not agree with the notion that vaccines are import, effective and safe. In this study about half of the other countries had even lower fractions of extremely vaccine hesitant people and thus Germany seems to be in the middle in a Western European comparison [22]. These studies indicate that, in general, vaccinations are relatively well accepted in Germany and it could be speculated that general extreme vaccine hesitancy might not be the main reason for the low uptake of HPV vaccines. And 3), the vaccine delivery system in Germany may be one likely very relevant reason for the low uptake of HPV vaccination in Germany. As described above, vaccinations are provided by office-based physicians and the physician or the parent needs to actively raise the need for HPV vaccination once the child becomes eligible making the system extremely opportunistic. There is no universal routine health care visit for adolescents that is generally used to administer the HPV vaccine. Effective means to improve the delivery structure could include the introduction of a vaccination register, an electronic vaccination card, recall systems and the introduction of school-based vaccination. School-based HPV awareness and vaccination campaigns are one of the measures that are also proposed by the German health ministers to improve HPV vaccination coverage and there are regional pilot school-based HPV vaccination programs implemented in Germany [10].

The decline in primary HPV vaccinations during the COVID-19 pandemic described here exacerbates the problem of low vaccination coverage and calls for additional measures. Models exist for the USA, where a similar decline was observed, after which several years of intensified vaccination activity would be required to make up the deficit [23], and many thousands of additional cases of HPV-associated diseases can be expected over a period of 100 years, depending on the recovery of vaccination rates [24].

In summary it can be concluded from our study that after an initial dynamic increase in HPV vaccinations in boys, the impact of COVID-19 was particularly strong in the second year of the pandemic. At the end of 2021 vaccination rates for boys were still at low level, which may be due to the still relatively short time since the recommendation in 2018 and universal reimbursement in 2019, as well as due to the COVID-19 pandemic. Efforts are needed to increase HPV vaccination rates in Germany and catch-up on adolescents that missed doses during the pandemic.

## Data Availability

The data set can be requested from the corresponding author.
